# Case Report: A novel *PHOX2B* p.Ala248_Ala266dup variant causing congenital central hypoventilation syndrome

**DOI:** 10.3389/fped.2022.1070303

**Published:** 2023-02-15

**Authors:** Irina N. Artamonova, Anna M. Zlotina, Olga R. Ismagilova, Tatyana A. Levko, Natalia Yu Kolbina, Aleksandr V. Bryzzhin, Andrey P. Smorodin, Alexandr V. Borodin, Ekaterina A. Mamaeva, Anna A. Sukhotskaya, Ilya M. Kagantsov, Daria A. Malysheva, Elena S. Vasichkina, Tatiana M. Pervunina, Natalia A. Petrova

**Affiliations:** ^1^ Institute of Perinatology and Pediatrics, Almazov National Medical Research Centre, Saint-Petersburg, Russia; ^2^ Institute of Molecular Biology and Genetics, Almazov National Medical Research Centre, Saint-Petersburg, Russia; ^3^ Federal State Budgetary Scientific Institution, Research Centre for Medical Genetics (RCMG), Moscow, Russia; ^4^ Department of Pediatric and Medical Rehabilitation, Almazov National Medical Research Centre, Saint-Petersburg, Russia; ^5^ Department of Pediatric and Medical Rehabilitation, Almazov National Medical Research Centre, Saint-Petersburg, Russia; ^6^ Pediatric Anesthesiology and Intensive Care Unit, Almazov National Medical Research Centre, Saint-Petersburg, Russia; ^7^ Pediatric Surgery Anesthesiology and Intensive Care Unit Almazov National Medical Research Centre, Saint-Petersburg, Russia; ^8^ World-Class Research Centre for Personalized Medicine, Research Centre of Unknown, Rare and Genetically Determined Diseases, Almazov National Medical Research Centre, Saint-Petersburg, Russia; ^9^ Institute of Perinatology and Pediatrics, Almazov National Medical Research Centre, Saint-Petersburg, Russia; ^10^ Department of Pediatric Surgery for Congenital Malformations, Almazov National Medical Research Centre, Saint-Petersburg, Russia; ^11^ Department of Pediatric Surgery for Congenital Malformations, Institute of Perinatology and Pediatrics, Almazov National Medical Research Centre, Saint-Petersburg, Russia; ^12^ Department of Pediatric Surgery for Congenital Malformations, Almazov National Medical Research Centre, Saint-Petersburg, Russia; ^13^ World-Class Research Centre for Personalized Medicine, Research Centre of Unknown, Rare and Genetically Determined Diseases, Almazov National Medical Research Centre, Saint-Petersburg, Russia; ^14^ Institute of Perinatology and Pediatrics, World-Class Research Centre for Personalized Medicine, Research Centre of Unknown, Rare and Genetically Determined Diseases, Almazov National Medical Research Centre, Saint-Petersburg, Russia; ^15^ World-Class Research Centre for Personalized Medicine, Research Centre of Unknown, Rare and Genetically Determined Diseases, Institute of Perinatology and Pediatrics, Almazov National Medical Research Centre, Saint-Petersburg, Russia

**Keywords:** genotype–phenotype correlation, congenital central hypoventilation syndrome (CCHS), *PHOX2B*, novel mutation, Hirschsprung disease, polyalanine sequence

## Abstract

**Introduction:**

Congenital central hypoventilation syndrome (CCHS) is a rare disease characterized by central alveolar hypoventilation and impaired autonomic regulation, caused by pathogenic variants of *PHOX2B* gene. More than 90% of patients have a polyalanine repeat mutation (PARM) in the heterozygous state, characterized by the expansion of GCN repeats and an increase in the number of alanine repeats, so that genotypes 20/24–20/33 are formed (the normal genotype is 20/20). The remaining 10% of patients harbor non-PARMs.

**Case description:**

We present a clinical case of a girl with a novel *PHOX2B* heterozygous genetic variant in the exon 3: NM_003924.4: c.735_791dup, p.Ala248_Ala266dup. The duplication includes 16 GCN (alanine) repeats and 3 adjacent amino acids. Both clinically healthy parents demonstrated a normal *PHOX2B* sequence. In addition, the girl has a variant of unknown significance in *RYR1* gene and a variant of unknown significance in *NKX2-5* gene. The child's phenotype is quite special. She needs ventilation during sleep, and has Hirschsprung's disease type I, arteriovenous malformation S4 of the left lung, ventricular and atrium septal defects, coronary right ventricular fistula, hemodynamically nonsignificant, episodes of sick sinus and atrioventricular dissociation with bradycardia, divergent alternating strabismus, and oculus uterque (both eyes) (OU) retinal angiopathy. Two episodes of hypoglycemic seizures were also registered. Severe pulmonary hypertension resolved after appropriate ventilation adjustment. Diagnostic odyssey was quite dramatic.

**Conclusion:**

Detection of a novel *PHOX2B* variant expands the understanding of molecular mechanisms of CCHS and genotype–phenotype correlations.

## Introduction

Congenital central hypoventilation syndrome (CCHS) is a rare disease characterized by central alveolar hypoventilation and impaired autonomic regulation. In most cases, patients have normoventilation during wakefulness and hypoventilation during sleep, most pronounced in the non-rapid eye movement (non-REM) sleep phase (1–3). The estimated incidence of the disease in population is 1:200,000 (4).

CCHS is associated with the pathogenic variants of the *PHOX2B* gene, which encodes a highly conserved homeobox transcription factor. As a result, the expression of a number of genes is altered, which leads to a disruption in the formation of the autonomic nervous system (5–9). More than 90% of patients have a polyalanine repeat mutation (PARM) in the heterozygous state, characterized by the expansion of GCN repeats and an increase in the number of alanine repeats, so that genotypes 20/24–20/33 are formed (the normal genotype is 20/20). The remaining 10% of patients harbor non-PARMs (NPARMs) including missense, frameshift, nonsense, and splice site variants in one of three *PHOX2B* exons. (10). Single cases of partial or whole-gene deletions were also described (11). Pathogenic variants in the *PHOX2B* gene are autosomal dominant and in most cases occur *de novo*; however, in 5%–10% of cases, asymptomatic mosaicism is detected in one of the parents (10). The direct correlation between the number of polyalanine repeats and the severity of the disease has been proposed (10, 12, 13). Hirschsprung’s disease (detected in 20% of patients, Haddad syndrome), neural crest tumors (neuroblastoma, ganglioneuroma), glucose metabolism disorders (hypoglycemia, signs of hyperinsulinism), as well as various signs of autonomic dysfunction (thermolability, arrhythmias, fluctuations in blood pressure, sudden episodes of muscle hypotension, various ophthalmic symptoms) are more common among 20/27–20/33 and NPARM genotypes. Patients with 20/24–20/25 PARM genotypes present with mild phenotypes and late-onset variants (10, 14–17).

We present a clinical case of a girl with a newly described *PHOX2B* variant including duplication of 16 GCN (alanine) repeats and 3 adjacent amino acids. The child's phenotype–genotype correlation is quite special.

## Case description

A 3.5-year-old girl freshly diagnosed with CCHS was transferred to our hospital for further evaluation.

The girl was born at term by cesarean section from healthy non-consanguineous parents. She developed respiratory insufficiency during the first hours of life requiring constant lung ventilation. Extubation attempts were unsuccessful due to bradypnea (10–15 breaths per minute), desaturations (SpO_2_ up to 60%–75%), and hypercapnia (values are not available) during sleep. After one of the extubations, lung bleeding occurred, and arteriovenous malformation S4 of the left lung was diagnosed on a CT scan. The girl developed clonic seizures at the age of 3 months, which resolved after phenobarbital administration. She required prolonged ventilation, extubation attempts were still unsuccessful, with an episode of cardiorespiratory arrest, and was tracheostomized at the age of 4 months.

Subsequently, being undiagnosed, the girl was not ventilated appropriately with long periods of self-breathing during sleep, which resulted in desaturations. Decannulation to mask ventilation at 36 months failed due to intolerance, followed by re-tracheostomy. The spontaneous breathing during sleep was insufficient; however, ventilation was still sporadic. The girl suffered frequent pneumonias and tracheitis with purulent bloody sputum.

On echocardiography, ventricular septal defect, 4 mm atrial septal defects, and coronary right ventricular fistula (CAF) were diagnosed, and considered hemodynamically nonsignificant. However, chronic heart failure developed: dilation of the right chambers of the heart, right ventricle hypertrophy, pulmonary hypertension (calculated systolic pressure in the right ventriculum 70 mmHg), hepatomegaly developed by the age of 18 months, and ejection fraction was 64%–72% by Teichholz. By the age of 42 months, ejection fraction decreased to 49% and ascites developed. Episodes of sick sinus and atrioventricular dissociation with bradycardia (37–51 beats per minute, pauses up to 2,255 ms) were first diagnosed at the age of 19 months. At further evaluations, heart rhythm improved; however, episodes of bradycardia during daytime persisted.

The girl suffered constipation since birth and megacolon or dolichosigma was suspected due to ultrasound and irrigography findings.

Apart from that, thrombocytopenia persisted (80–124 × 10 × 9/L at 12–36 months).

At the age of 42 months, first episode of hypoglycemic seizures took place (blood glucose level 1.38 mmol/L, sodium 120 mmol/L, and chloride 77 mmol/L).

Genetic test was done only at the age of 3.5 years. Initially, a blood sample of the patient was sent to a commercial genetic testing laboratory for whole-exome sequencing (WES) analysis. WES was carried out using SureSelect All Exon V7 target enrichment kit (Agilent Technologies, CA, United States) and Illumina NovaSeq 6000 instrument with average target region coverage of ∼170× (98.8% of targeted nucleotides with coverage >10×). The laboratory provided us with a report with WES results including genetic variants possibly related with the clinical phenotype and secondary incidental findings in genes recommended by the ACMG (18). Based on WES data, the girl has a missense variant of uncertain significance—chr19:g.38993563 G>C, NM_000540.3:c.7879G>C (p.Val2627Leu) (rs914804033)—in *RYR1* gene, in which pathogenic variants are known to be associated with malignant hyperthermia susceptibility (OMIM # 145600) but seemingly not with a CCHS condition. Moreover, WES allowed us to reveal a rare genetic variant in cardiac homeobox gene NKX2-5: chr5:g.172661909 C>G, NM_004387.4: c.178G>C, (p.Glu60Gln), (rs766199339). Notably, no *PHOX2B* variations were mentioned in the report.

Then, *PHOX2B* sequencing was performed in the Research Centre for Medical Genetics, Moscow; afterward, results were validated in our institution by bidirectional Sanger sequencing. The sequencing procedure was carried out using the BigDye Terminator Sequencing Kit (Applied Biosystems) and Genetic Analyzer AB3100 (Applied Biosystems/Hitachi, Japan). The primers were designed using the NCBI Primer Blast tool (Gene ID: 8929, NG_008243.1; exon 1: F 5′-AATTTTGTTGGCGGTTCGGG-3′, R 5′-TAGGCTCTGCTGGTAGTAAGGA-3′; exon 2: F 5′-AATCCAGTATTTCTGATCGGCCA-3′, F 5′-TGAAAGCACTATCTCAAGTCCGT-3′; exon 3a F 5′-CATACTGCTCTTCACTAAGGCG-3′, R 5′-GAGGGTGTTAAAACAAGCCGA-3′; exon 3b F 5′-GGCCCTCAATGAAAAAGCCA-3′, R 5′-TCCTCGGGCAAAAAGTCTGA-3′).

**Figure 1:**
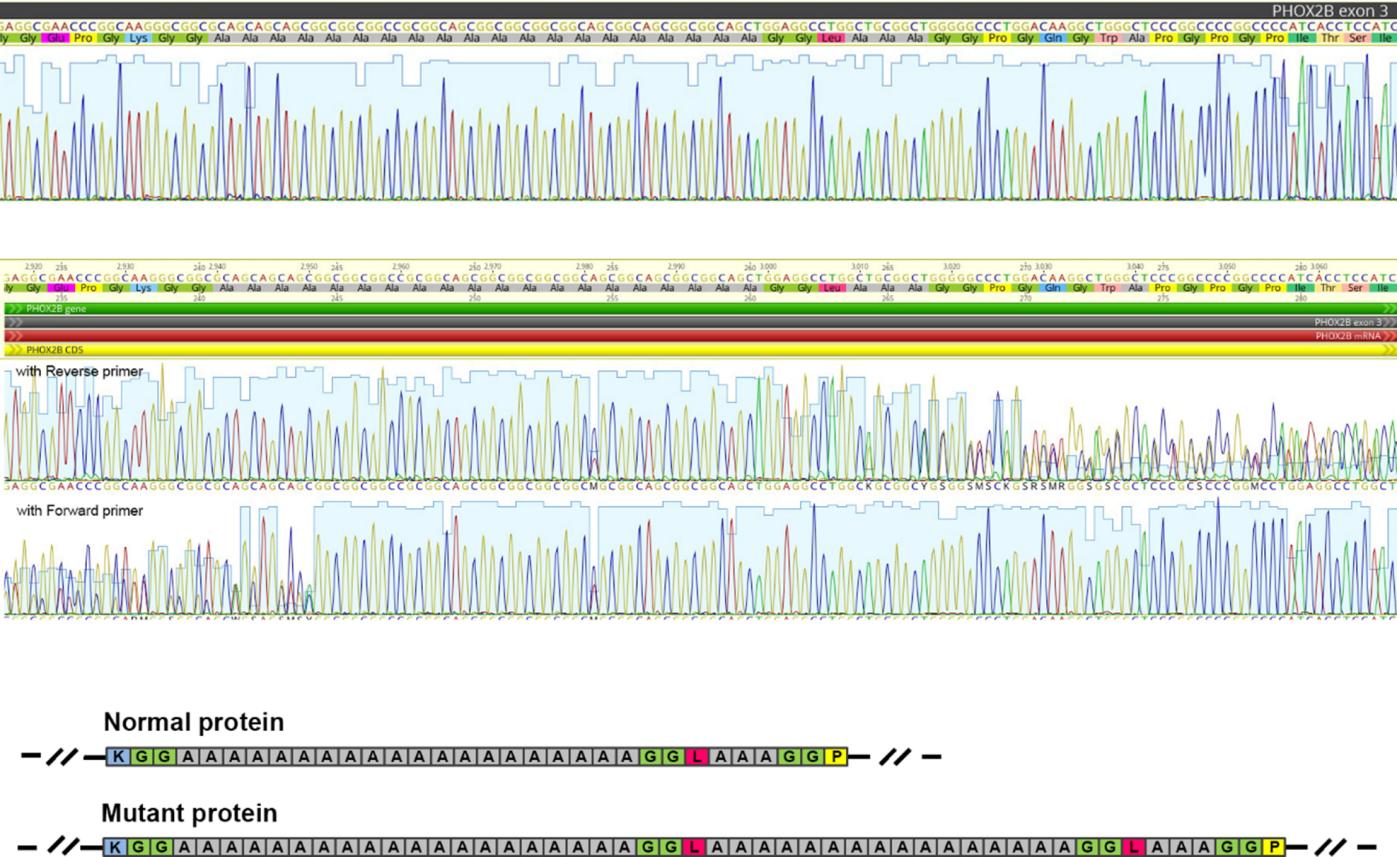
The *PHOX2B* genetic variant (p.Ala248_Ala266dup) was revealed by Sanger sequencing. The figure shows a fragment of Sanger sequencing chromatogram corresponding to the polyalanine repeat and flaking regions in a control (“reference”) sample (**A**) and in the reported patient (**B**). (**C**) The scheme illustrating the impact of the duplication on the amino acid sequence in the mutant protein. Colored boxes represent amino acid residues (blue—lysin, green—glycine, grey—alanine, magenta—leucine, and yellow—proline).

Target sequencing of PHOX2B protein-coding regions allowed us to identify a novel heterozygous genetic variant in the exon 3: NM_003924.4: c.735_791dup, (p.Ala248_Ala266dup) (Figure 1). The 57-bp duplication corresponds to 13 GCN (alanine) repeats plus 6 adjacent amino acids (Gly-Gly-Leu-Ala-Ala-Ala). It represents a non-frameshift duplication leading to the protein elongation (+19 amino acids).

Both clinically healthy parents demonstrated a normal *PHOX2B* sequence.

After the diagnosis had been made at the age of 44 months, the girl was transported to our hospital. By admission, she was ventilated through a tracheostomy tube 3–5 h per night followed by awakening and subsequent unsuccessful attempts to resume ventilation caused by overventilation in REM sleep. The girl's height was 87 cm (−3.14 SD), weight was 11 kg (−2.55 SD), and weight-to-height ratio was −0.89 SD. Respiratory support was adjusted under tcCO_2_ monitoring: ST rate 25/min, Pi 15 cm Н_2_О, Pimax 21, EPAP 5 сm Н_2_О, FiO2-21%, Tin-0,7 s. The vital need for mechanical ventilation during sleep was explained to the parents.

Echocardiography showed muscular ventricular septal defect (2 mm), atrial septal defect (2–3 mm), ejection fraction of 62.5% (by Teichholz), and systolic pulmonary artery pressure of 36 mmHg. On 48-h Holter ECG, heart rate appeared normal, but there was sinus arrhythmia with pauses up to 1,248 ms, QTc prolongation up to 511 ms, and a decrease in heart rate variability with no nighttime increase in the high-frequency component of variability. Ophthalmologic examination discovered divergent alternating strabismus OU and retinal angiopathy OU.

Neurologic status was as follows. The girl maintained head upright all the time, sat and stood without support, walked independently well, kicked a ball forward, and threw a ball over hand. The girl could not run, jump, and walk upstairs. Her understanding of the addressed speech was full. She used pincer grasp, held the pen, but could not copy shapes (circle, square, etc.) or imitate vertical line. Denver Developmental Screening test at age of 4 years showed MQ = 0.62 (N ≥ 0.75) and DQ = 0.57 (N ≥ 0.7). Her cranial innervation was intact. She had muscle hypotonia, with muscle strength of 5 points in limbs according to the MRС scale (Medical Research Council scale for power of muscle), valgus flat foot. Tendon reflexes were normal. There were no meningeal and cerebral symptoms.

**Figure 2:**
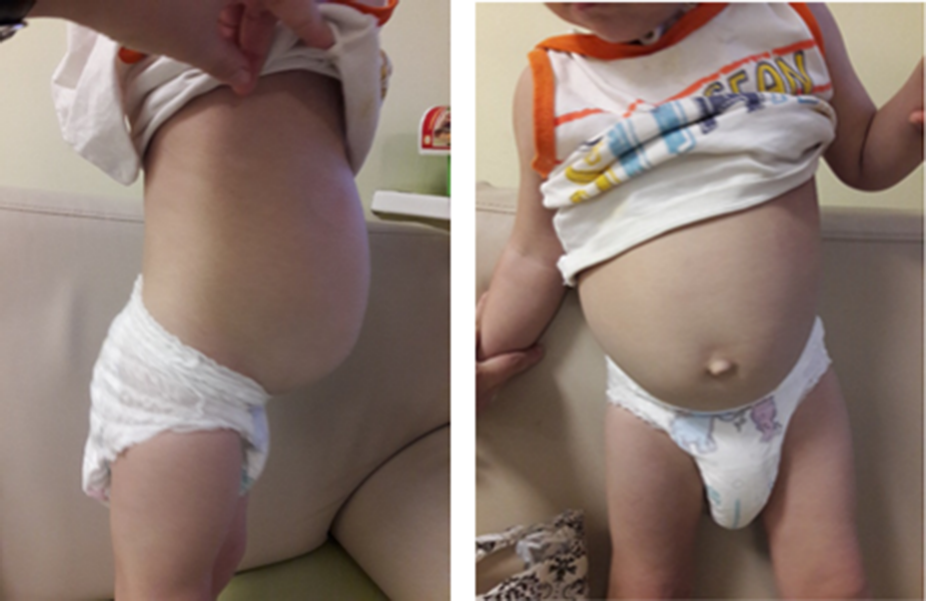
Hirschsprung's disease: abdomen distension.

**Figure 3:**
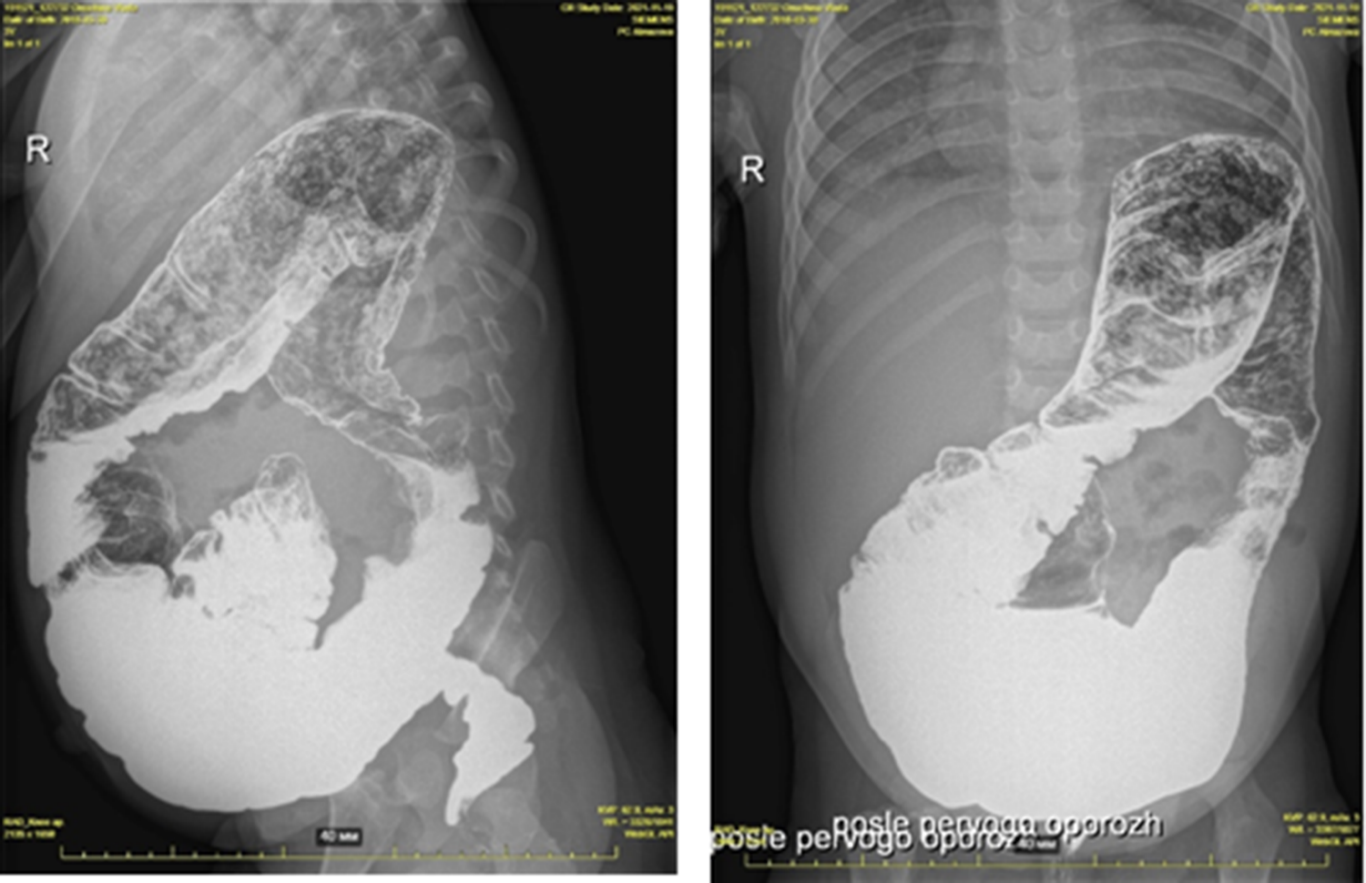
Hirschsprung's disease. Barium enema showing reduced caliber of the rectum, followed by a transition zone to an enlarged-caliber sigmoid.

Hirschsprung's disease was suspected due to chronic constipation, an increase in the volume of the abdomen (Figure 2). Irrigography showed narrowing of the rectum and sigmoid colon with pronounced suprastenotic expansion (Figure 3).

A laparoscopy with a biopsy of the colon was performed, histology showed aganglionosis, which indicates Hirschsprung’s type I disease. A colostomy was done on the descending colon. After the surgery, bloating decreased, and ventilation improved.

Five months later, the girl was readmitted. Her height was 89 cm (−3.21 SD), weight was 14.25 kg (−0.9 SD), and weight-to-height ratio was +1.67 SD. LS Swenson pull-through was performed. After surgery, the ventilation settings were adjusted with the lower settings needed during REM sleep. Heart rate normalized with maximum QTc of 470 ms. Echocardiography data slightly improved: ejection fraction: 64.6% (by Teichholz) and systolic pulmonary artery pressure: 25 mmHg.

Another hypoglycemic episode took place at 48 months of age (glucose 2.1–2.35 mmol/L without electrolyte disorders). Afterward, glucose levels were kept under dynamic control; no more hypoglycemic episodes were registered.

**Figure 4:**
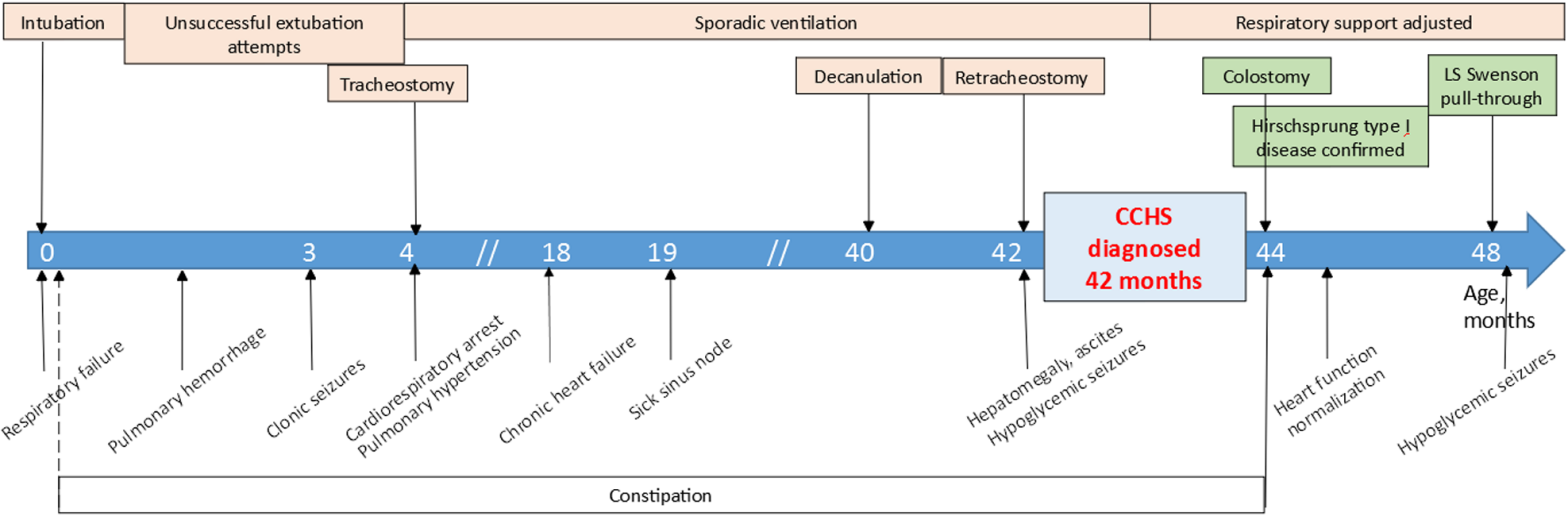
The disease course of the patient.

The disease course is showed in Figure 4.

## Discussion

CCHS is generally associated with PARM and NPARM pathogenic variants in *PHOX2B* gene (19). In the clinical case reported here, we revealed a novel heterozygous variant (p.Ala248_Ala266dup) in the exon 3 of *PHOX2B*. Notably, the initial whole-exome sequencing analysis did not reveal this genetic variation. Such results are in accordance with the accumulated data pointing to ineffectiveness of new generation sequencing (NGS) approaches such as targeted gene panel sequencing and exome sequencing for polyalanine repeat expansion detection (20). Here, we once again confirm that Sanger sequencing is an adequate and relevant tool for detection of genetic variants affecting *PHOX2B* polyalanine repeat region, which is necessary for early and correct diagnosis of CCHS conditions.

As for classification of the genetic variant, it is not so straightforward. Taking into account its size and content, the duplication should not be regarded as a traditional PARM, although it overlaps with the polyalanine repeat region. Anyway, the genetic variant results in a change in the extent and structure of the polyalanine repeat region, apparently leading to the altered protein organization. Previously, functional effects of missense, frameshift, and alanine expansion pathogenic variants in *PHOX2B* sequence were carefully evaluated (21). Trochet et al. showed the declined transactivation of the dopamine beta-hydroxylase promoter (a direct *PHOX2B* transcriptional target) by *PHOX2B* with polyalanine expansions. In addition, DNA binding was consistently decreased in the longest expansions (+9 alanines and above). Interestingly, the authors tested not only traditional expansions ranging from +5 to +13 alanines but also an alanine expansion interrupted by a threonine residue (Ala)_11_(Thr)(Ala)_15_. Such a variant demonstrated transactivation and DNA binding properties within the normal range (21). It implies that different kinds of alterations in the polyalanine repeat region could have varying impact on *PHOX2B* protein function, and, therefore, on the severity of the clinical phenotype. Further *in silico* and/or *in vitro* functional studies could specify in more detail the molecular consequences of the (p.Ala248_Ala266dup) variant.

*PHOX2B* is essential for normal function of autonomous nervous system (22), and hence all autonomic processes.

In PARMs, the severity of the disease almost always depends on the number of GCN repeats, with 20/24–20/26 genotypes presenting with mild hypoventilation and respiratory support during sleep only, while in patients with 20/27–20/33 genotypes, ventilation requirements may include daytime needs and vary up to 24/7 ventilation. These high-grade PARMs are also associated with higher Hirschprung's disease (HD), autonomic nervous system dysfunction (ANSD), and neural crest tumor (NCT) risk (10, 19, 23–25). Few exceptions were presented so far: Kasi et al. described a late-onset case with 20/27 genotype (26), while Lee-Kelland et al. could not find significant correlation between the length of GCN expansion and minute ventilation during either REM or non-REM sleep (27). NPARMs, on the other hand, are not so straightforward (24, 28). They are in general considered more pathogenic than PARMs and are highly associated with HD and NCT (10, 28, 29). However, there are several reports of NPARM genotypes with relatively mild phenotypes (28, 30, 31). It was speculated that the severity of NPARM depends on the *PHOX2B* exon with severe variants linked to exon 3, while pathogenic variants in exon 1 present with mild phenotype (23). Some cases of NPARM as well as 20/24 PARM ones present as late-onset CCHS in adolescence or even adulthood or even have no clinical signs at all (28, 32). Zhou et al. supposed that exon 1 and 2 loss-of-function NPARM variants activate nonsense-mediated mRNA decay (NMD), leading to absence of the truncated protein, like full-gene deletions do, and thus resulting in more mild phenotype (12). Exon 3 variants, being in the last exon of the *PHOX2B* gene, are less likely to trigger NMD control pathway, so the truncated protein is produced, having a potentially detrimental dominant-negative effect (12).

Of rare cases, compound heterozygous and homozygous variants were described (28). However, to our knowledge, there are no descriptions of such atypical PARM cases with prolonged GCN repeats interrupted by additional non-alanine amino acids.

As mentioned above, children with high-grade PARMs are supposed to have severe hypoventilation and prolonged need for ventilation which was not the case in our patient, who required respiratory support only during sleep.

From the study by Trochet et al., we can speculate that interruption of polyAla tract by different amino acids may have “protective” effect on phenotype (21). While Trochet et al. divided polyalanine expansion into two short parts (11 and 15 alanines), which led to normalization of transactivation and DNA binding properties, in our case we have a prolonged interrupted expansion of 20 + 16 alanines and a phenotype of nighttime (mostly non-rapid eye movement (NREM)) hypoventilation, which is not as severe as could be expected, in combination with HD, and autonomic dysregulation signs: sick sinus and atrioventricular dissociation with bradycardia, and divergent alternating strabismus (21). Further *in silico* and/or *in vitro* functional studies could specify in more detail the molecular consequences of the variant. The effect of expanded polyAla tract interruption on clinical presentation could be confirmed if other similar cases were described.

The multisystem presentation of CCHS requires a multidisciplinary approach. Apart from the evident need of respiratory support and regular respiratory physician's consultations with polysomnography and/or blood gas evaluations, full assessment must include testing of ANSD (neurologist, ophthalmologist, fasting, and postprandial glucose monitoring) and cardiac evaluation (including Holter monitoring, echocardiography, and arterial blood pressure measurement) (33–37). In 20/27-20/33 PARM, and NPARM genotypes oncology search is required (chest radiography, abdomen ultrasound, and brain MRI in some cases) (19). This approach was first proposed in 2010 by Weese-Mayer et al. (10) and further revised in 2020 (38) and 2022 (19).

Cardiovascular abnormalities usually described in CCHS patients are sinus node dysfunction, QTc elongation, and blood pressure dysregulation due to ANSD. Hypoventilation and hypoxia result in pulmonary hypertension and cor pulmonale (32). In our patient, apart from arrhythmia and pulmonary hypertension, we saw multiple structural defects: small septal defects, CAF, and pulmonary arteriovenous malformation (PAVM) S4 of the left lung. Association of CCHS and congenital heart defects (CHDs) was shown in two reports. Lombardo et al. reviewed single-institution population of CCHS patients and found a 30% (6/20) prevalence of CHD. CHDs were found in five children with NPARMs (multiple atrial septal defect (ASD), patent ductus arteriosus, vertebral, coronary arteries anomalies, complete vascular ring), and a secundum ASD in a patient with a 20/33 PARM (39). Similar prevalence of CHD was found in the study of Mei et al.; however, exact CHD types were not provided and patent ductus arteriosus and atrial septal defect were prevalent (40).

At the same time, WES revealed a rare genetic variant in cardiac homeobox gene *NKX2-5*: chr5:g.172661909 C>G, NM_004387.4: c.178G>C, (p.Glu60Gln), (rs766199339). To our knowledge, this variant had not been previously reported as a genetic cause of any congenital heart disease cases in the literature. It is present in population allele frequency databases (GnomAD 0.03%), and there are contradictory computational predictions regarding its effect on protein structure and function. Thus, currently available data are insufficient to specify the contribution of the *NKX2-5* p.Glu60Gln variant to the patient's cardiac phenotype, and according to ACMG (41), it should be classified as a variant of unknown significance (VUS).

Unusual for CCHS, vascular anomalies were found in our patient: CAF, PAVM, and retinal angiopathy. It is known, that CAF and PAVM (42) are hereditary in up to 90% of cases and are often associated with telangiectasia (43), but such pathogenic variants were not discovered in our patient, as well as telangiectasia. Many cases of CAF and PAVM are asymptomatic (43, 44). The complications of PAVM may include hypoxemia, hemoptysis, pulmonary hypertension, high-output heart failure, and cerebrovascular accidents (42), but hypoventilation was not described. At that point, we believe that structural cardiovascular abnormalities in our patient do have genetic origin, but we cannot definitely attribute them to either *PHOX2B* or *NKX2-5*.

Based on WES results, the patient has a genetic variant c.7879G>C (p.Val2627Leu), rs914804033, in *RYR1* gene. The *RYR1* gene encodes the skeletal muscle ryanodine receptor playing a role of a calcium release channel of the sarcoplasmic reticulum and ensures connection between the sarcoplasmic reticulum and transverse tubule. To date, a spectrum of different autosomal dominant and recessive clinical conditions has been described in association with *RYR1* genetic variants. In particular, heterozygous, homozygous, or compound heterozygous *RYR1* variants can cause central core disease (OMIM #117000). Biallelic variants were also revealed in cases of minicore myopathy with external ophthalmoplegia (OMIM #255320). Susceptibility to malignant hyperthermia is another autosomal dominant skeletal muscle disorder caused by heterozygous pathogenic variants in *RYR1* (OMIM #145600). In addition, heterozygous *RYR1* variants can be a genetic basis of the King–Denborough syndrome (KDS), a clinical condition characterized by the triad of congenital myopathy, dysmorphic features, and susceptibility to malignant hyperthermia (45). The revealed *RYR1* genetic variant is present in population allele frequency databases (GnomAD Genomes *f* = 0.0000319), and it has been reported in individuals with a history of a malignant hyperthermia reaction. Our patient did not show clinical signs of neuromuscular disease. In her neurological state, we noted muscle hypotonia, most likely central, but there were no signs of muscle weakness.

Hypoglycemia in our case is consistent with well-described spectrum of glucose metabolism disturbances in CCHS (34) with no phenotype–genotype correlation.

The diagnosis and treatment of patients with CCHS are quite complicated and may be accompanied by late diagnosis of Hirschsprung's disease (10). The inclusion of a pediatric surgeon in a multidisciplinary team is important for appropriate diagnosis and treatment of HD. In the case we describe, HD was not diagnosed in time. We believe that all children with CCHS and constipation should be screened for HD, as well as all patients with HD and respiratory issues should be screened for CCHS.

Neurodevelopmental delay is an issue in CCHS patients. Its causes are disputable with input of recurrent or acute hypoxemia and hypoventilation, on the one hand, and *PHOX2B* dysfunction itself, on the other hand (19). Neurologic consequences may vary from severe headache and daytime drowsiness to status epilepticus (32).

Presence of mild cases along with mosaicism (both somatic and germinogene) and incomplete penetrance makes it necessary to examine all the relatives of CCHS patients.

In our case, in the course of diagnostic odyssey while hypoventilation was not appropriately adjusted, it resulted in heart failure, pulmonary hypertension, and arrhythmia, which at that time were addressed not to respiratory dysfunction but to heart and vascular structural anomalies (septal defects and coronary right ventricular fistula). A significant improvement was achieved after respiratory support correction, which makes us speculate that it was hypoventilation that caused these cardiovascular consequences. However, with late diagnosis, the changes may be irreversible (32).

## Conclusion

The detection of a novel *PHOX2B* variant expands the understanding of molecular mechanisms of CCHS and genotype–phenotype correlations.

Our case seems to combine clinical features of both nonsevere PARMs and NPARMs: the girl has HD, ANSD, and structural cardiovascular abnormalities, like severe NPARM cases do (24, 40, 46), but her hypoventilation is relatively mild, requiring non-invasive lung ventilation (NLV) only during sleep. It was proposed that *PHOX2B* pathogenic variants have variable penetrance depending on unknown epigenetic factors (28, 46), and further evaluation is needed. The understanding of molecular effects of different kinds of alterations in the polyalanine repeat region could provide more information on estimated phenotype changes. Association of *PHOX2B* pathogenic variants with cardiovascular anomalies needs extra research. The diagnostic odyssey also illustrates an insufficient awareness of CCHS and pathophysiology of autonomic regulation disorders among medical society.

## Data Availability

The original contributions presented in the study are included in the article/Supplementary Material, further inquiries can be directed to the corresponding authors.
